# Personalized auricular vagus nerve stimulation: beat-to-beat deceleration dominates in systole-gated stimulation during inspiration - a pilot study

**DOI:** 10.3389/fphys.2024.1495868

**Published:** 2025-01-06

**Authors:** Johannes Tischer, Jozsef Constantin Szeles, Eugenijus Kaniusas

**Affiliations:** ^1^ Institute of Biomedical Electronics, Vienna University of Technology, Vienna, Austria; ^2^ Center for Wound Surgery and Special Pain Therapy, Health Service Center, Wiener Privatklinik, Vienna, Austria

**Keywords:** neuromodulation, auricular vagus nerve stimulation, heart rate variability, cardiac-gated stimulation, respiratory-gated stimulation, personalized stimulation

## Abstract

Neuromodulation comes into focus as a non-pharmacological therapy with the vagus nerve as modulation target. The auricular vagus nerve stimulation (aVNS) has emerged to treat chronic diseases while re-establishing the sympathovagal balance and activating parasympathetic anti-inflammatory pathways. aVNS leads still to over and under-stimulation and is limited in therapeutic efficiency. A potential avenue is personalization of aVNS based on time-varying cardiorespiratory rhythms of the human body. In the pilot study, we propose personalized cardiac-gated aVNS and evaluate its effects on the instantaneous beat-to-beat intervals (RR intervals). Modulation of RR is expected to reveal the aVNS efficiency since the efferent cardiac branch of the stimulated afferent vagus nerve governs the instantaneous RR. Five healthy subjects were subjected to aVNS. Each subject underwent two 25-min sessions. The first session started with the non-gated open-loop aVNS, followed by the systole-gated closed-loop aVNS, then the non-gated, diastole-gated, and non-gated aVNS, each for 5min. In the second session, systole and diastole gated aVNS were interchanged. Changes in RR are analysed by comparing the prolongation of RR intervals with respect to the proceeding RR interval where aVNS took place. These RR changes are considered as a function of the personalized stimulation onset, the stimulation angle starting with R peak. The influence of the respiration phases is considered on the cardiovagal modulation. The results show that the systole-gated aVNS tends to prolong and shorten RR when stimulated after and before the R peak, respectively. The later in time is the stimulation onset within the diastole-gated aVNS, the longer tends to be the subsequent RR interval. The tendency of the RR prolongation raises with increasing stimulation angle and then gradually levels off with increasing delay of the considered RR interval from the one where aVNS took place. The slope of this rise is larger for the systole-gated than diastole-gated aVNS. When considering individual respiration phases, the inspiratory systole-gated aVNS seems to show the largest slope values and thus the largest cardiovagal modulatory capacity of the personalized time-gated aVNS. This pilot study indicates aVNS capacity to modulate the heartbeat and thus the parasympathetic activity which is attenuated in chronic diseases. The modulation is highest for the systole-gated aVNS during inspiration.

## 1 Introduction

Neuromodulation, particularly the electrical stimulation of the autonomic nervous system, has emerged as a promising non-pharmacological therapeutic approach for various chronic ailments (Guiraud et al., 2016; Edwards et al., 2017; González-González et al., 2024). The electrical vagus nerve stimulation presents an appealing target for neuromodulation since the vagus nerve connects mutually the brain and the body, not only governing but also sensing vital physiological processes within the heart, lungs, and gastrointestinal tract (Câmara and Griessenauer, 2015).

Invasive and non-invasive methods exist for the vagus nerve stimulation, with the non-invasive methods gaining popularity due to lower risk and cost profile (Ben-Menachem et al., 2015; Redgrave et al., 2018; Kaniusas et al., 2019a). A non-invasive auricular vagus nerve stimulation (aVNS) has emerged (Yap et al., 2020) as a safe treatment option (Kim et al., 2022) for the different disorders (Kaniusas et al., 2019a; Verma et al., 2021; Gerges et al., 2024), such as atrial fibrilliation (Stavrakis et al., 2015), migraine (Grazzi et al., 2018), chronic pain (Szeles et al., 2021), depression (Hein et al., 2012), and respiratory ailments including severe cases in COVID-19 (Seitz et al., 2022). aVNS alters signal processing and activates reflex circuitries in the brain, exploits brain plasticity and neural adaptation, affects nociceptive processing and parasympathetic anti-inflammatory pathways, with the modulation of the brain chemistry and autonomic function leading to re-established sympatho-vagal balance with reduced sympathetic dominance, especially, in chronic diseases (Bonaz et al., 2016; Butt et al., 2019; Kaniusas et al., 2019a).

Despite promising results of aVNS, the reported therapeutic outcomes lack consistency and are difficult to predict; the required therapeutic dose is unknown, with potential side effects due to over and under-stimulation that generate non-responders (Farmer et al., 2021; Ottaviani et al., 2022). The stimulation parameters are empirically selected while the stimulation strength is usually titrated based on one-time individual perception of the patient (Warren et al., 2019). This open-loop aVNS disregards momentary and time-varying therapeutic needs in view of the individual and time-varying physiological state of the patient, which, in fact, is the very target of aVNS treatment.

A closed-loop aVNS, i.e., a continuously personalized aVNS, with an instantaneous and continuous biofeedback is required to account for the individual physiology (Yu et al., 2022). aVNS settings are continuously adapted based on the biofeedback and subsequently avoid, for instance, over and under-stimulation. The biofeedback is given by various autonomic biomarkers such as cardiac cycle and heart rate variability, respiration cycle, saliva composition, pupil diameter and reflex, and electroencephalography (Warren et al., 2019; Konakoğlu et al., 2023; Bömmer et al., 2024). For instance, the onset of closed-loop aVNS has been synchronized with the oromotor activity of neonates during feeding using electromyography as biofeedback (Badran et al., 2020) or synchronized with task-specific training in post-stroke rehabilitation (Badran et al., 2023), improving respectively sensorimotor skills in neonates with oromotor deficits or motor function in stroke survivors, known as motor-activated aVNS. However, a recent review by (Soltani et al., 2023) highlighted that the physiological response of biomarkers such as standard measures of the heart rate variability to aVNS remains inconsistent. This is due to methodological limitations of the standard non-instantaneous analysis of the short-term heart rate variability (Task Force of the European Society of Cardiology and the North American Society of Pacing and Electrophysiology, 1996) when using non-stationary segments of heartbeat intervals of variable duration with uncontrolled respiration rate, due to heterogeneous and incomparable aVNS setting across studies with patient-specific baselines as well as to a lack of understanding of mechanistic principles of the closed-loop aVNS (Fan et al., 2024).

Not only magnitude-related settings of aVNS can be customized (e.g., stimulation magnitude) but also time-gated aVNS settings (e.g., onset and offset of aVNS) (Kaniusas et al., 2019b; Thompson et al., 2021; Mylavarapu et al., 2023). Therefore, the temporal stimulation sequences of aVNS are synchronized with inner biological rhythms of the body to interfere with residual activities of the autonomic nervous system in favour of therapy. Electroencephalography (EEG) is used as biofeedback signal in the closed-loop EEG-gated aVNS, with the stimulation onset targeting the rising delta phase during non-rapid eye movement sleep; the EEG-gated aVNS aims to modulate the delta power of EEG, thereby modulating arousal and reducing neuroinflammation, both of which affect delirium as a therapeutic goal (Anzolin et al., 2023). A prominent example is given by the respiratory-gated aVNS (García et al., 2022; de Faria et al., 2024): aVNS is paced with the expiration phase when the brainstem, namely, the nucleus of the solitary tract, affected by rhythmical modulations with the respiration rate and where most afferent fibers of the vagus nerve terminate (Berthoud and Neuhuber, 2001; Karemaker, 2022), is expected to be more receptive to the afferent vagal input. Finally, a cardiac-gated aVNS, namely, ECG-gated aVNS, has been suggested as time-gated aVNS in future (Yu et al., 2022) because aVNS has already shown promising applications in cardiovascular field (Kaniusas et al., 2019a) and electrocardiogram (ECG) is considered as reliable tool as biofeedback signal for monitoring heart diseases.

Cardiorespiratory coupling, especially the respiratory sinus arrhythmia (RSA), is important in terms of the gated aVNS, which is vagally mediated and shortens heartbeat intervals during inspiration and lengthens them during expiration (Yasuma and Hayano, 2004). RSA arises in the course of various phenomena, such as (i) direct commands from respiration centres in the brain (pacing vagal neurons with the respiration rate), (ii) baroreflex control (co-determining the vagal outflow to the heart as a function of the arterial blood pressure, with stronger influence of baroreceptors during expiration), (iii) the Bainbridge reflex (the right atrial volume or the central venous pressure governs the vagal outflow), and (iv) other central reflexes involving chemoreceptors (in the carotid bodies) and mechanoreceptors (in the lungs) (Kaniusas, 2012). RSA is a measure of the autonomic regulation, often referred to as “cardiac age”, and is impaired in chronic disease. Considering the above, in our opinion, synchronous cardiac and respiratory dynamics have to be taken in account in the personalised setting the parameters of the aVNS practice.

We propose and evaluate cardiac-gated aVNS and its instantaneous non-averaged modulatory effects on the cardiovagal branch of the heart, governed by the autonomic nervous system. In particular, we investigate these modulatory effects of the systole-gated aVNS (i.e., with aVNS onset at the start of the cardiac systole) and the diastole-gated aVNS (i.e., with aVNS onset at the start of cardiac diastole) on beat-to-beat intervals. These intervals are triggered by the cardiovagal branch projecting from the brain to the sinoatrial node, the pacemaker of the heart. Moreover, we consider explicitly the respiration phase of the applied aVNS and thus the respiration influence on heartbeats. To the best of our knowledge, this is the first report on cardiac-gated aVNS, namely, systole-gated or diastole-gated aVNS, compared with non-personalized aVNS, with considered respiration phases.

## 2 Methods

### 2.1 Experimental data

A pre-clinical pilot study was conducted to investigate the physiological response of the cardiac-gated aVNS. This single-center study was approved by the institutional review board of the Medical University of Vienna (EK NR 2121/202, entitled: “Cardiac-gated auricular vagus nerve stimulation in a closed-loop biofeedback system, for adaptive stimulation in healthy subjects - pilot study”). Five healthy subjects (2 female) aged 30.8±10.3 years were subjected to the percutaneous auricular VNS while monitored via ECG and the respiration belt (sampling frequency of 1 kHz).

**FIGURE 1:**
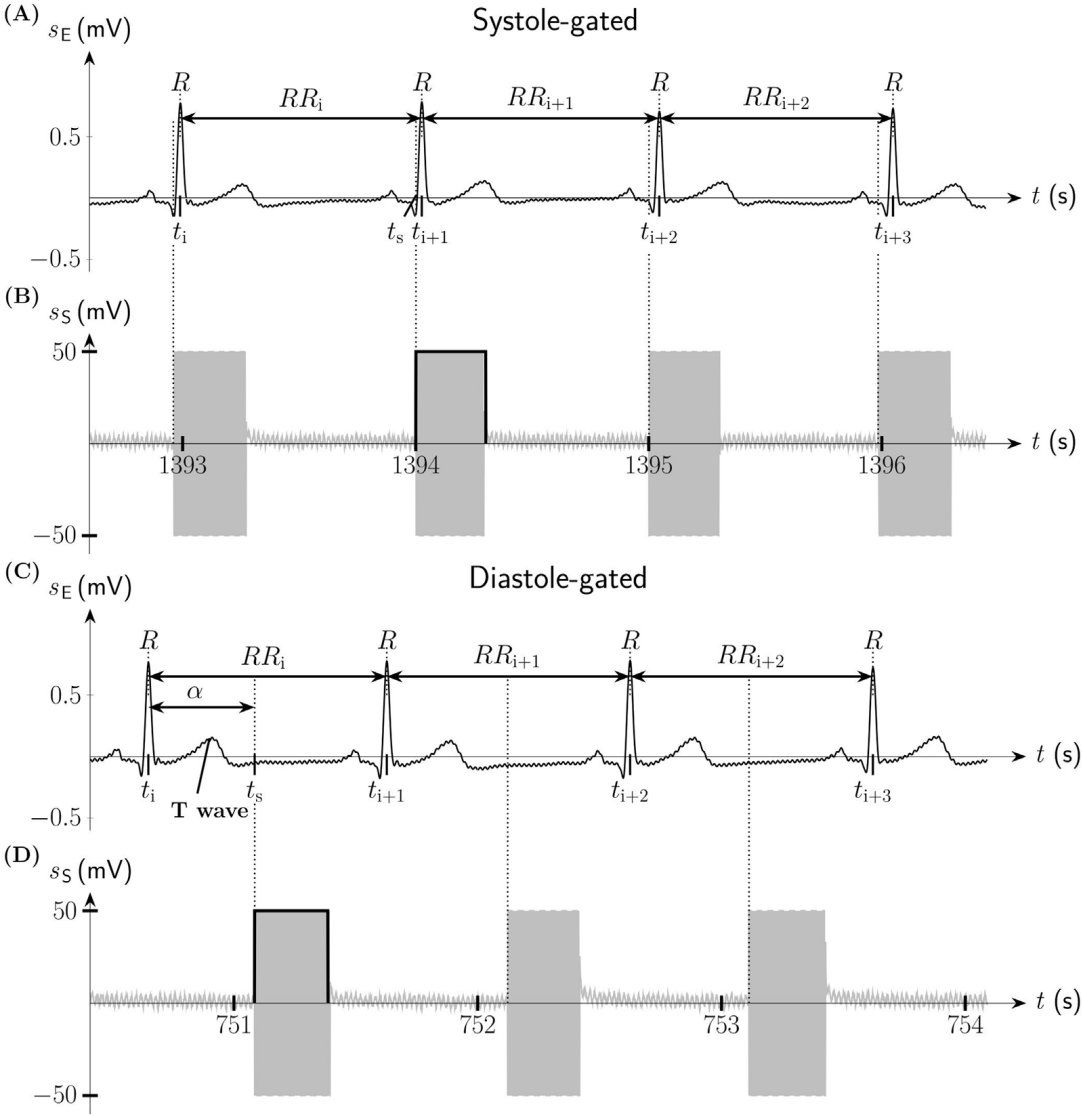
Systole-gated and diastole-gated auricular vagus nerve stimulation (aVNS). **(A)** Electric biosignal electrocardiogram $s_{E}$ (lead I Einthoven) with indicated R peaks and the associated RR intervals during systole-gated aVNS. **(B)** Systole-gated aVNS with the stimulation signal $s_{S}$ triggered by the onset of the systole of the heart at the time point ***t*_S_**. **(C)** $s_{E}$ during diastole-gated aVNS. The timing of aVNS in respect to the R peaks is delineated by the stimulation angle α . **(D)** Diastole-gated aVNS with $s_{S}$ triggered by the onset of the diastole of the heart.

For aVNS, tripolar stimulation was performed with three miniature stimulation needles placed in the left ear, each with a penetration depth of 1–2 mm. Auricular regions were selected for stimulation, which are partly or solely innervated by the vagus nerve: cymba conchae and cavity of conchae (Peuker and Filler, 2002). Needles were positioned close to vessel-nerve bundles, as located by transillumination of the auricle (Kaniusas et al., 2011) and by visual inspection of the auricular vessel structure. Needle electrodes were wired with the voltage-controlled output of a proprietary portable μC-based stimulator, build by the Vienna University of Technology (Dabiri et al., 2022). Three different triphasic stimulation patterns were applied on the three needle electrodes, each composed out of six consecutive pulses of different magnitudes of 500µs duration each, with the total duration of 3 ms of a single triphasic symbol, for details see (Kaniusas et al., 2019c). The sum over all three stimulation patterns equals to zero voltage at any time, making obsolete any reference electrode and increasing robustness of aVNS. A fixed burst length of 100 triphasic symbols per stimulation event was implemented, with the resulting burst duration of 300 ms per stimulation event; see Figure 1 for demonstrated successive bursts with the magnitude of 50 mV.

Each subject underwent two sessions on different days but at the same time of the day (to minimize circadian influence) in a quiet room and sitting position. The session started after a setup phase, ensuring resting pulse, resting respiratory rate, and at least 2min accommodation to aVNS after placement of needle electrodes. The stimulation magnitude was increased from 0 mV in steps of 100 mV to reach a clear tingling perception - as reported by the subject - but not pain, and then was fixed for the whole session. After each session, needles were removed. The electrode position was slightly altered from the first to second session in order to avoid both formation of scar tissue and increase in the electrode impedance.

Each session lasted for 25min. The first session consisted of a subsequent application of the non-gated open-loop aVNS (stimulation bursts applied every second) for 5min, the systole-gated closed-loop aVNS (stimulation burst onsets within the systolic phase, starting with the R peak in ECG) for another 5min, then again the non-gated open-loop aVNS for 5min, followed by the diastole-gated closed-loop aVNS (stimulation burst onsets within the diastolic phase derived from ECG) for 5min, and finally the non-gated aVNS for the last 5min. In the second session, the systole-gated and diastole-gated intervals were interchanged.

### 2.2 Closed-loop aVNS

The R peak derived from ECG - used as biofeedback for the closed-loop aVNS - served as a reference point within the cardiac cycle for the time-gated aVNS. Since the real-time closed-loop aVNS stimulator has intrinsic delays in the range of 100 ms (Dabiri et al., 2022) for the processing of ECG and formation of stimulation patterns for the output of the closed-loop aVNS, an instantaneous cardiac-gated aVNS is not possible at an arbitrary time point within the cardiac cycle of the just detected R peak. Thus, a simple predictive stimulation was realized for the next heart cycle in which the stimulation took place.

Namely, the associated five consecutive beat-to-beat intervals were monitored, covering approximately the last full respiration cycle. The average of these intervals, assuming that the subjects were in their resting stationary state, allowed to estimate the duration of the following heartbeat interval and thus the approximate position of the next predicted R peak. In this interval, the cardiac-gated stimulation could be triggered at any time point within the cardiac cycle, in spite of the aforementioned delays. The resulting uncertainty in this simple prediction was quantitatively monitored and evaluated via histograms.

**FIGURE 2:**
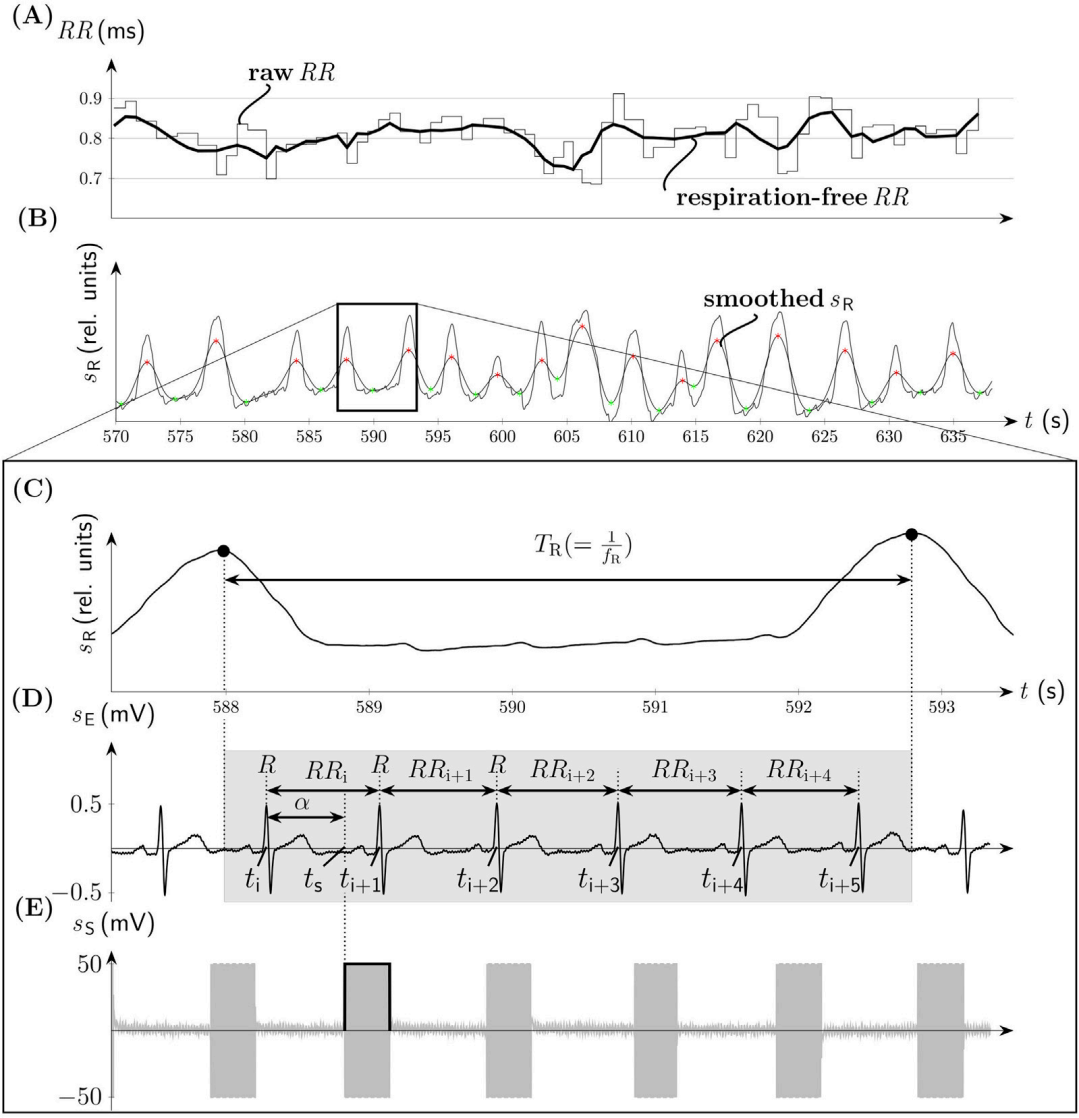
Non-gated aVNS (compare Figure 1). **(A)** The time series of RR intervals with its filtered respiration-free version (thick solid line). **(B)** The associated respiration signal $s_{R}$ with the onset of the inspiration (green) and expiration (red) phases. **(C)** The zoomed version of $s_{R}$ with the indicated respiration period $T_{R}$ . **(D)** Electrocardiogram $s_{E}$ with denoted RR intervals at time points $t_{i}$ , $t_{i+1}$ ,…, $t_{i+5}$ and the onset of the stimulation with the angle α at the time point ***t*_S_**. The duration of the respiration cycle is indicated by the gray background. **(E)** The associated bursts of aVNS.

Thus, the systole-gated aVNS was realized at the predicted R peak following the detected R peak. Figures 1A, B illustrates the onset of the systole-gated aVNS with the R peak at the time point *t*_S_. For the diastole-gated aVNS, aVNS starts 500 ms after the predicted R peak to match approximately the end of the T-wave of ECG (i.e., the start of diastole), as illustrated in Figures 1C, D. The choice of 500 ms delay lies in the assumption that the diastole begins at rest after about 40% of the cardiac cycle duration (the cycle starting with the R peak), so that for the expected cycle duration of about 1s at rest (and shorter cycles), the proposed delay of 500 ms should guarantee the onset of the diastole-gated aVNS within the initial section of diastole. In contrast, the non-gated aVNS, as shown in Figures 2D, E, shows no synchrony with the ECG waveform, with *t*_S_ periodically occurring with 1Hz in both systole and diastole.

### 2.3 Data processing

The recorded beat-to-beat intervals (*RR* intervals, the distance between two consecutive R peaks) were detected from ECG using proprietary algorithms (based on peak detection tools) and visually controlled for absent ectopic beats and strong movement artefacts. In total, 8811 heartbeats (subject 1-5: 2093, 1819, 1707, 1689, 1503) were included finally in the beat-to-beat analysis. The data processing was conducted with MATLAB R2023b (The MathWorks Inc., MA).

In order to quantify the prediction with the monitored aVNS patterns and ECG, an accurate stimulation location is reconstructed within each individual cardiac cycle. All cycles are normalized to 360° and start at the R peak (i.e., located at 0° or 360°), which allows a joint consideration of heartbeats of different individual durations. This normalization maps the duration of the cardiac cycle onto a scale from 0° to 360°, with values exceeding 360° corresponding seamlessly to values greater than 0°, that captures the repetitive nature of the cardiac cycle.

The respective stimulation angle α within the cardiac cycle - as demonstrated in Figure 2D - is defined as

$$\alpha =\left(\frac{t_{S}-t_{i}}{t_{i+1}-t_{i}}\right)\cdot 360{}^{\circ}$$ (1)

where $t_{i+1}$ is the measured time point of the R peak after aVNS, $t_{i}$ the measured time of the R peak before the stimulation, and $t_{S}$ the measured time of the onset of the burst of aVNS (considering variable delays of the stimulator). Consequently, the systole-gated aVNS is theoretically described by $\alpha \approx 0^{\circ }$ (Figure 1A) while the diastole-gated aVNS by a theoretical $\alpha \approx 180^{\circ }$ (Figure 1C).

The physiological impact of the cardiac-gated aVNS is beat-to-beat evaluated considering for each *RR* interval in which aVNS takes place, the duration modification in up to seven subsequent cardiac cycles. Specifically, considering the *i*-th heartbeat with aVNS onset, i.e., *RR*_i_,

$$RR_{i+n}=t_{i+1+n}-t_{i+n},$$ (2)

the percentual change ΔRR _i+1+n_ of a subsequent *RR*_i+1+n_ is calculated as:

$$\Delta RR_{i+1+n}=\frac{RR_{i+1+n}-RR_{i}}{RR_{i}}\cdot 100\;\%$$ (3)

where i = 1,2, … N-7, n = 0,1, … 6, and N is the number of heartbeats analysed (Figure 2D).

Then, the change $\Delta RR_{i+1+n}$ is evaluated as a function of α (based on the measured times from Equation 1) in scatterplots, for each considered heartbeat in the non-gated and gated aVNS. A linear regression is calculated in scatterplots using equal α ranges for the systole-gated aVNS ( α = 0° ± 60°), the diastole-gated aVNS ( α = 180° ± 60°), and for the non-gated aVNS ( α = 0°–360°). The slope of the linear regression line is then determined for each subject, yielding five slopes per stimulation protocol and per ΔRR _i+1+n_.

For RSA-based investigations, we pre-filter *RR* intervals with a FIR band-stop filter (Kaiser-Bessel window, filter order 52) with the cut-off frequency situated at the respiration rate *f*_R_. This diminishes the respiration-related changes in *RR* intervals and thus attenuated RSA effects (Figure 2A). Before the application of this filter, *RR* intervals were linearly interpolated with 3Hz to arrive at equidistant time points. The instantaneous *f*_R_ is derived from the respiration belt signal, subjected first to moving average filter to smooth it (Figure 2B). Then onsets of inspiration and expiration phases are acquired as minima and maxima, respectively, of the smoothed respiration signal for each individual respiration cycle while using proprietary algorithms (based on the first derivatives). These onsets enable finally the calculation of *f*_R_ (Figure 2C). The pre-filtered version of *RR* is referred to as respiration-free *RR*. The calculus of $\Delta RR_{i+1+n}$ is performed on both, the raw *RR* and respiration-free *RR* sequences. The detected onsets of inspiration and expiration phases were also used to allocate the onsets of the applied individual aVNS bursts either to inspiration or expiration. This allows to conduct the linear regression analysis (from above) separately for inspiration and expiration phases of individual respiratory cycles.

## 3 Results

The personalized aVNS, the systole-gated and diastole-gated aVNS, are illustrated in Figure 1. The stimulation bursts onset with the measured stimulation angle α that is close to 0° for the systole-gated aVNS; i.e., aVNS starts with the R peak, with the start of systole (Figure 1A). For the diastole-gated aVNS, the angle α is close to 180°; i.e., aVNS starts after the T wave in ECG (Figure 1C). By contrast, the non-personalized non-gated aVNS - as shown in Figures 2D, E − shows dispersed values of α between $0^{\circ }$ and $360^{\circ }$ , with stimulation bursts being out of synchrony with R peaks of ECG. The raw and respiration-free *RR* sequences (Figure 2A) are illustrated along the respiration signal and its smoothed version (Figure 2B).

**FIGURE 3:**
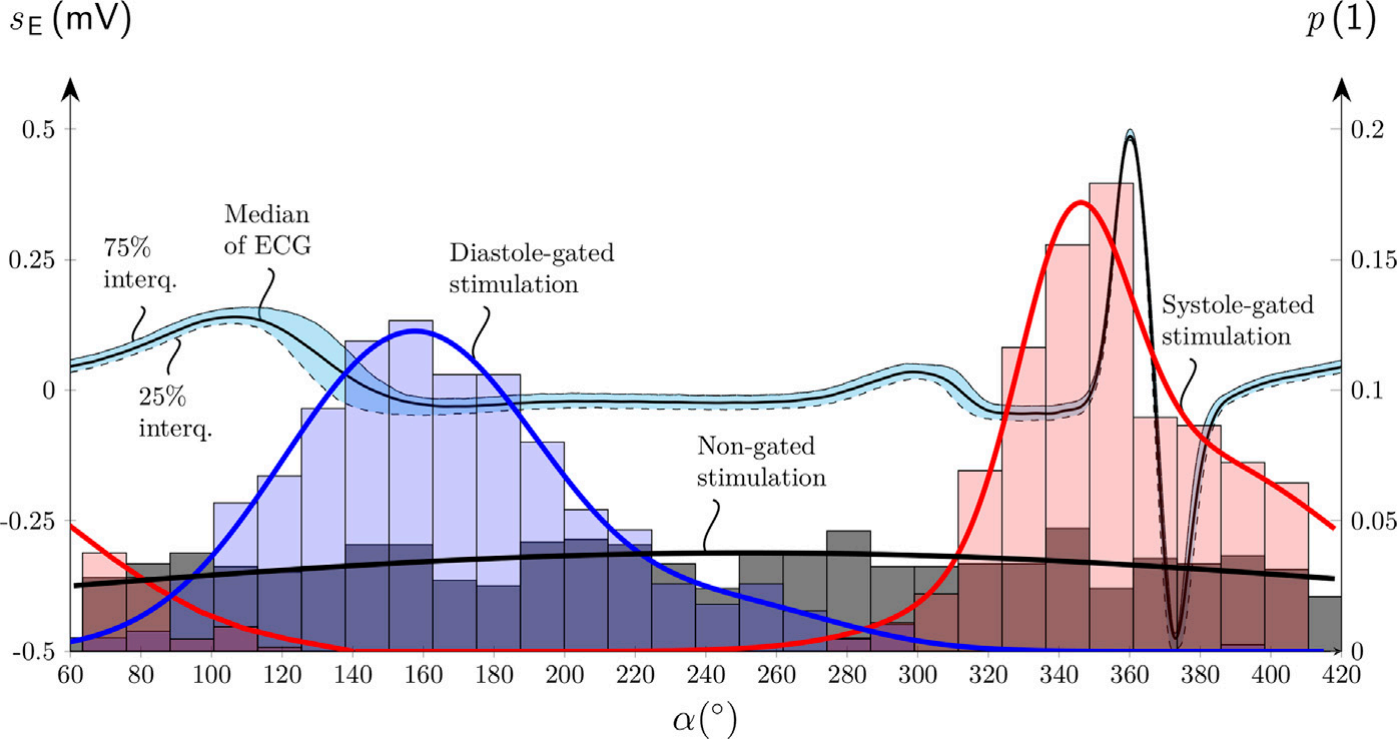
Histograms of the onset of the aVNS bursts with their probability p of the systole-gated (red), diastole-gated (blue), and non-gated (black) aVNS as a function of the stimulation angle α aligned along the normalized electrocardiogram $s_{E}$ cycle for a single subject (compare Figure 1). Median and interquartile curves of $s_{E}$ are shown, as well as Gaussian fits of all three histograms.

The dispersions of the measured α at the targeted α=0° for the systole-gated aVNS and at the targeted α=180° for the diastole-gated aVNS are quantitatively assessed via histograms to evaluate if the measured α coincides with the theoretical 0° and 180° for the two aVNS settings, as shown in Figure 3. In contrast to the non-gated aVNS with α equally distributed in the range from 0° to 360°, the systole-gated aVNS exhibits a maximum probability around at $0^{\circ }$ while the diastole-gated aVNS peaks near $160^{\circ }$ . For the systole-gated aVNS, the targeted and mean actual values of α overlap ( α = 0°) with a dispersion of about ±20% at 50% height of the histogram. For the diastole-gated aVNS, the targeted and mean actual values of α do not overlap ( α = 180° versus 160°), with even a larger dispersion of about ±35% at 50% height.

**FIGURE 4:**
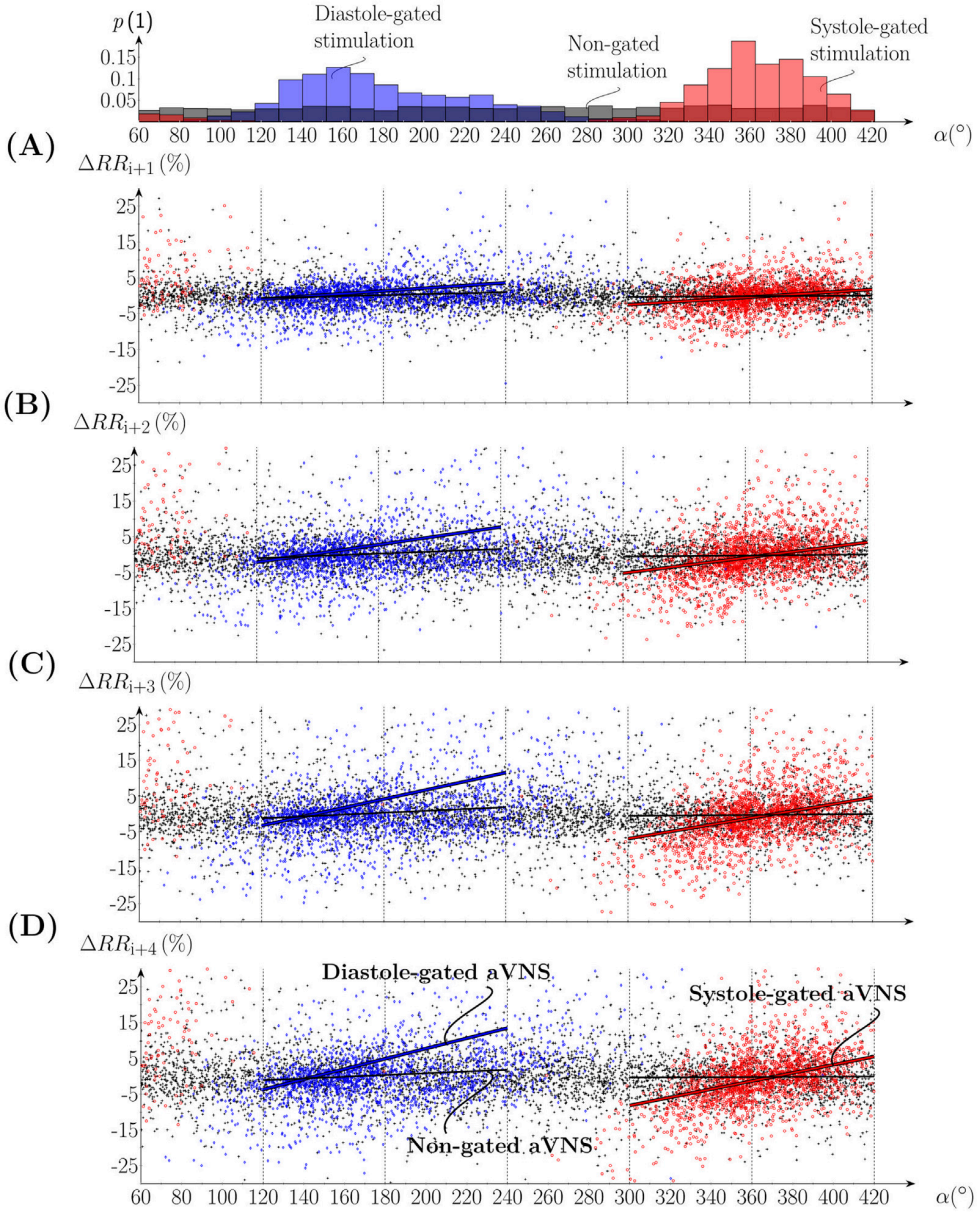
Scatterplots of the percentual change of **(A-D)** ${RR}_{i+1}$ to ${RR}_{i+4}$ in relation to ${RR}_{i}$ , where the onset of aVNS occurred, as a function of the stimulation angle α of all subjects. The top subfigure shows the associated histograms of systole-gated (red), diastole-gated (blue), and non-gated (black) aVNS; compare Figure 3. Linear regression fits are given for gated aVNS (solid red and blue lines) and non-gated aVNS (black lines).

Figure 4 shows scatterplots of $\Delta RR_{i+1+n}$ (Equation 3) with n = (0, 1, 2, 3) as a function of α for all subjects; i.e., it considers four consecutive *RR* intervals (Equation 2) following the *RR* interval with the active aVNS (the considered *RR* intervals as depicted in Figure 2D). The indicated slope of the linear regression line tends to increase from n = 0 to n = 3 for both the systole-gated and diastole-gated aVNS. By contrast, zero slope results for the non-gated aVNS. The crossing point of the regression line with the horizontal line at ΔRR=0 (i.e., no relative change in *RR*) is at about α=11° for the systole-gated aVNS (10.7°, 11.7°, 11.6°, and 11.6° for n = (0, 1, 2, 3)) and at about 145° for the diastole-gated aVNS (145.3°, 145.8°, 146.0°, and 145.7° for n = (0, 1, 2, 3)).

**FIGURE 5:**
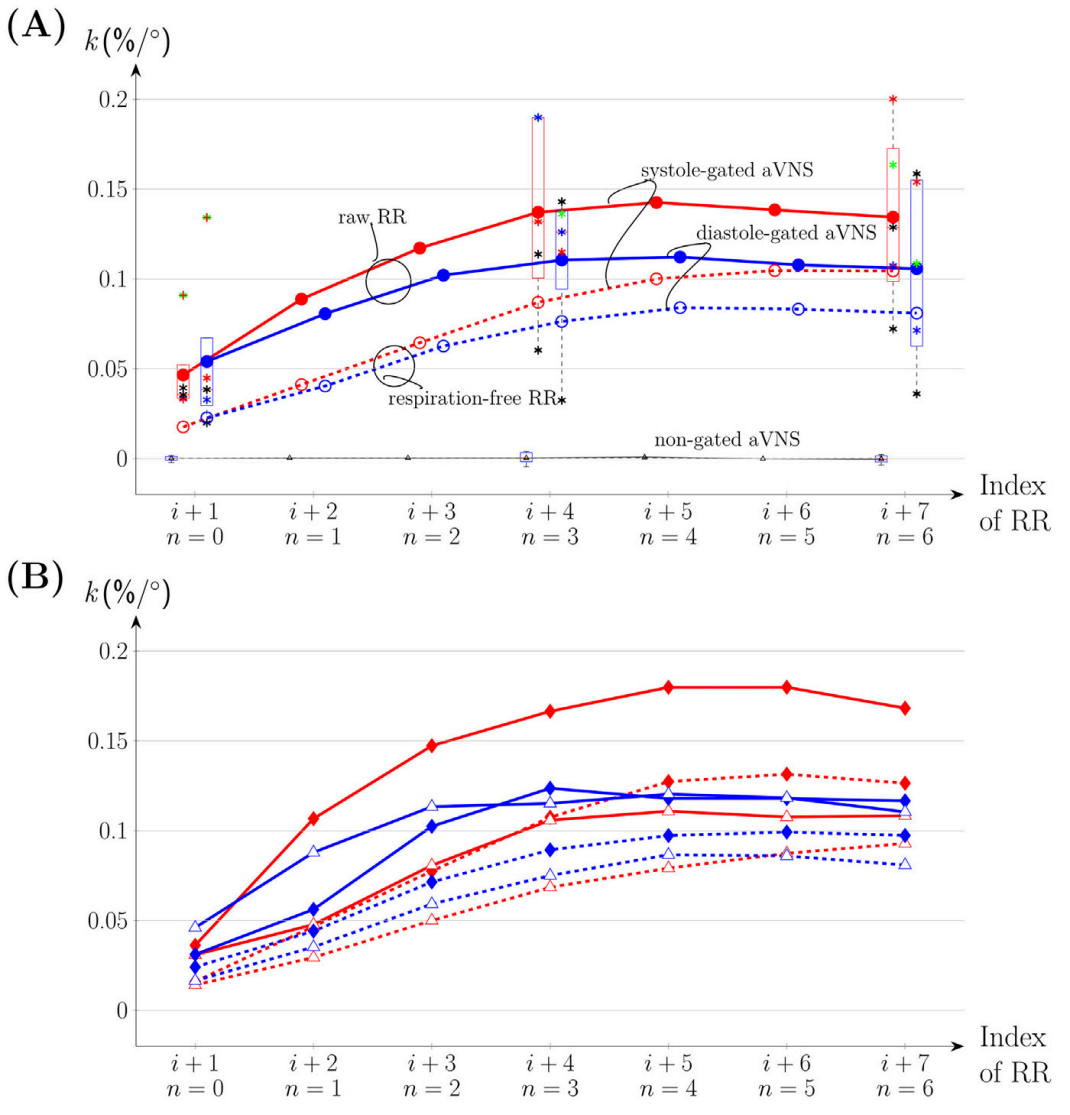
The slope k of the linear regression fit for ${∆RR}_{i+1+n}$ and n = 0,1, … 6 visualized for systole-gated (solid red connecting means for all subjects), diastole-gated (solid blue), and non-gated aVNS (solid black) for all subjects, including *k* values for the filtered respiration-free version of ***RR*** (dashed lines). **(A)** The whole respiration cycle. Boxplots are included with *k* values of individual subjects for n = 0, 3 and 6. **(B)** Inspiration (♦) versus expiration (△).

**FIGURE A1:**
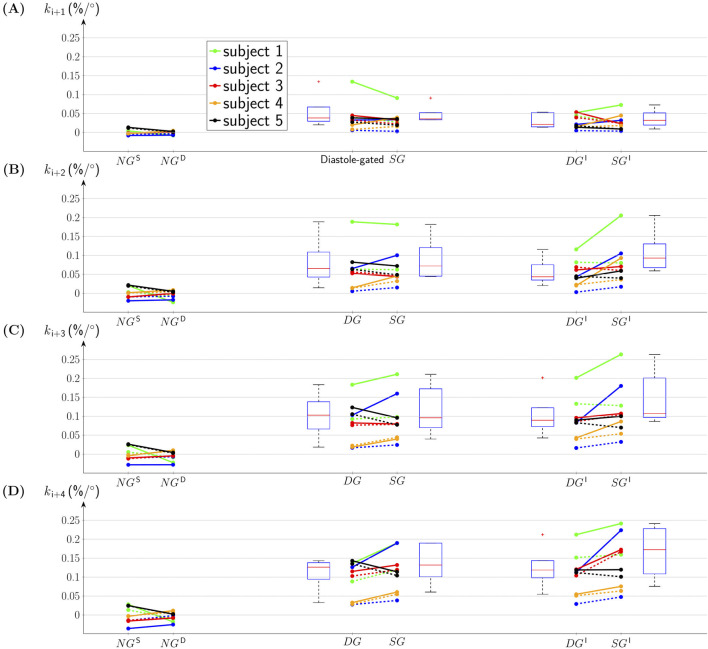
The slope k of the linear regression fit for **(A–D)** ${∆RR}_{i+1+n}$ and n = 0,1,2,3 visualized for non-gated aVNS (NG), diastole-gated aVNS (DG), and systole-gated aVNS (SG) for each subject. The first column presents *k* values during NG, including systole and diastole phases of the heart (for α from −60° to 60° for the systole phase and from 120° to 240° for the diastole phase). The second column displays *k* values during DG and SG, considering both inspiration and expiration phases. The final two columns show *k* values separate for inspiration (index I) and expiration (index E). Respective boxplots illustrate distribution of *k* across each stimulation protocol.

Figure 5 illustrates the slopes of the linear regression lines for n = (0, 1, … 6) as the mean value for all subjects (connecting lines in Figures 5A, B) and individual values for n = 0, 3, and 6 (boxplots in Figure 5A). That is, seven consecutive RR intervals are considered, including gated and non-gated aVNS. With increasing n, the mean values of the gated aVNS increase and then the y level off at about the fourth heartbeat (n = 3) following the one with the active aVNS. The mean slopes for the systole-gated aVNS start at 0.047%/° (n = 0) and end up at 0.134%/° (n = 6), while those for the diastole-gated aVNS start at 0.054%/° (n = 0) and end up at 0.106%/° (n = 6) (Figure 5A); for individual changes in the slope see Figure A1. By contrast, the non-gated aVNS shows the mean slope of zero (1.3e-5%/° for n = 0 and 3.7e-4%/° for n = 6). When the respiration-free *RR* sequence is used as a basis for the slope calculus, the mean slope values decrease by an offset of about 0.025%/° for all n values, as compared with the unfiltered raw *RR* (Figure 5A). The mean slope values for the respiration-free *RR* reach 0.105%/° (n = 6) for the systole-gated and 0.08%/° (n = 6) for the diastole-gated aVNS.

Figure 5B considers separately inspiration and expiration phases for all subjects with individual data provided in Figure A1. The maximum mean values of the regression slope for all n are observed for the systole-gated aVNS during inspiration, with the peak value of about 0.18%/° at n = 4. In relative terms, for the systole-gated aVNS, inspiration increases the mean slope by >0.05%/° for n > 0 and the observed ΔRR by even about 50% in relation to expiration. In contrast, the diastole-gated aVNS shows similar mean values of the slope for all n regardless of the respiration phase. The respiration-free RR decreases the respective slopes for the systole-gated and diastole-gated aVNS during both inspiration and expiration.

## 4 Discussion

The time-gated aVNS, namely, the systole-gated and diastole-gated aVNS, is realized to evaluate its effects on the efferent cardiovagal branch of the autonomic nervous system that governs heartbeats. The resulting beat-to-beat RR intervals are assessed to derive their individual duration changes. These beat-to-beat modulatory effects are expected to disclose efferent vagus nerve modulation by aVNS that stimulates the afferent vagus nerve in the ear.

Our hypothesis is that the systole-gated aVNS is superior to the diastole-gated aVNS. Since most of the afferent vagal inflow from arterial baroreceptors into the nucleus of the solitary tract is present in systole (Karemaker, 2017), this may indicate that the brainstem could be more receptive in systole for the artificially-induced afferent vagal inflow from aVNS. Consequently, the resulting modulation efficiency of the personalized time-gated aVNS (Figure 1) is quantified and also compared with that of the non-personalized non-gated aVNS (Figure 2).

The realized systole-gated and diastole-gated aVNS show the onset of the periodic stimulus aligned distinctly with the onset of systole and diastole, respectively (Figure 1). This alignment is completely random in the non-gated aVNS (Figure 2). The stimulation angle α - introduced in this work - quantifies jointly the systole-gated, diastole-gated, and non-gated aVNS. For the time-gated aVNS, the R peak in ECG served as the reference point of the current cardiac cycle. Given a non-zero processing time of ECG and intrinsic delays in the output formation of aVNS (Dabiri et al., 2022), a flexible time-gated stimulation can only be performed within the next cardiac cycle. This requires prediction of the next R peak to target the associated systole or diastole, the start of which initiates the systole-gated and diastole-gated aVNS, respectively. The attained prediction accuracy is reflected by the position and dispersion of histograms in Figure 3. The limited accuracy is mainly due to (i) a predictive nature of the R peak detection, (ii) the ongoing RSA, periodically accelerating and decelerating RR intervals, with this respiration influence not considered explicitly by the prediction, and (iii) the natural and non-paced respiration of subjects during the study, rendering the respiratory modulation of RR intervals even more unpredictable. The accuracy is lower for the prediction of diastole than systole, which could be attributed to the fact that RSA mainly affects the duration of diastole but not that of systole (Kaniusas, 2012). The limited accuracy of the systole-gated aVNS (Figure 3) may also cause the stimulation onset before the R peak which would imply the end of diastole but not the start of systole. Similarly, the limited accuracy of the diastole-gated aVNS may result in stimulation at the end of systole. This may have compromised targeted modulation effects and indicates the need for an improved prediction algorithm with a narrower dispersion width of histograms (Figure 3).

The results show that the systole-gated aVNS tends to prolong and shorten the following RR intervals when stimulation started after and before the R peak, respectively. This can be derived from positive values of the slope and the crossing point (near α=11° of the regression lines in Figure 4) located close to the R peak, the physiological onset of systole (situated at α=0° , see Figure 3). This observation is especially dominant for later heartbeats starting approximately with the fourth heartbeat (with a larger slope, compare Figure 4A with Figure 4D) after the considered heartbeat with the active aVNS (Figure 5). This delayed accumulation of deceleration and acceleration of heartbeats (in response to aVNS) may be attributed to processing in the brain, for the brain separates the afferent vagus nerve (subject to electrical stimulation) and the efferent cardiovagal nerve (connecting to the sinoatrial node and thus governing RR intervals, subject to monitoring). From experimental physiology, cardiovagal responses are reported to have a relatively short delay of a few heartbeats only (Hartikainen et al., 1998; Feher, 2012) supporting the hypothesis of the processing delays.

In the diastole-gated aVNS, the later in time is the stimulation onset within diastole, the longer tends to be the subsequent RR intervals. This observation is based on the positive regression slope and the crossing point near α=145° (Figure 4), the angle which corresponds roughly to the physiological onset of diastole (Figure 3). The tendency of the RR prolongation (i.e., the regression slope) raises with increasing time distance (or delay) from the considered heartbeat where aVNS took place. Then, as in the case of the systole-gated aVNS, the increases in the slope gradually levels off at about the fourth heartbeat (Figure 5).

The regression slope is larger for the systole-gated than diastole-gated aVNS, especially for later heartbeats by about 25%, and is zero for the non-gated aVNS (Figure 5A). This would imply that the systole-gated aVNS modulates the duration of heartbeats stronger than the diastole-gated aVNS. From a physiological perspective, the systole-gated afferent stimulation of the vagus nerve in the ear coincides in time with afferent responses of baroreceptors in systole (Karemaker, 2017; Karemaker, 2022), which project also via the vagus nerve into the brainstem, the nucleus of the solitary tract. Thus, we hypothesise that this temporal coincidence raises the brain alertness to all vagal inflows during systole, including aVNS during systole, to minimize average metabolic needs of the brain.

We expect that aVNS may also influence *RR* intervals through the mechanism of RSA, in that the vagally-mediated aVNS may modulate the dominance of the vagally-mediated RSA (Yasuma and Hayano, 2004). When the respiration influence is filtered out of RR sequence, lower regression slopes result for the time-gated aVNS (Figure 5). Therefore, it may be hypothesised that modulatory effects of the time-gated aVNS are partly hidden within the respiration-related changes in RR and these modulatory effects could be related to RSA mechanisms in the brain, with the involved respiration centre in the medulla. When considering individual respiration phases, the systole-gated aVNS with the onset in inspiration shows largest regression slopes for all heartbeats (Figure 5B), e.g., larger by about 50% than in expiration. Thus, the inspiratory systole-gated aVNS seems to have the largest cardiovagal modulatory capacity. This is in contrast to the respiratory-gated aVNS claimed to be superior in expiration with respect to autonomic modulation (García et al., 2022; Napadow et al., 2012), as based on the idea that inspiration inhibits the activity of the vagus nerve so that aVNS may be more effective in expiration due to potentially increased brain perception to the afferent inflow induced by aVNS. However, this comparison is of limited validity since the authors did not synchronize their aVNS with the cardiac cycle. This disagreement may highlight the relevance of the momentary phase of the cardiac cycle on the modulatory capacity of the gated aVNS; all the more, for the diastole-gated aVNS, there is no obvious influence of the respiration phase. Cardiovagal effects of the systole-gated aVNS appear to be more dependent on the respiration cycle.

The presented pilot study has several limitations. Low number of volunteers were included who, in addition, were healthy and relatively young, thus not representing typical aVNS patients, aged and with chronic complaints. The gated and non-gated aVNS were interchanged every 5min, which is relatively short for the systemic autonomic effects to develop; in future, up to 15min intervals should be considered (Soltani et al., 2023). However, the total duration of sessions applying different aVNS settings in series should not be too long to avoid fatigue effects on the autonomic nervous system. The realized simple prediction holds true only if RR interval is subjected to a slight and slow variation. This is not the case, even though prediction accuracy was monitored by histograms (Figure 3), especially due to the natural and non-paced breathing of the subjects during the study.

The present study warrants further research on the cardiac-gated aVNS, with the focus on optimisation of heartbeat prediction considering explicitly the respiration influence (with the temporal resolution of the RSA dynamics) and using larger and more homogeneous study samples. In particular, advanced prediction of systole and diastole should be considered in future (Zacarias et al., 2024) and/or the use of an inverse of Equation 1, which imposes target values for the stimulation angle.

## 5 Conclusion

This pilot study indicates, for the first time, that a specific timing of aVNS within the cardiac cycle tends to influence the response of the autonomic nervous system, especially of the efferent cardiovagal branch of the system. The resulting modulation of the heartbeat duration indicates a favourable aVNS influence on the parasympathetic activity that is pathologically attenuated in chronic diseases. The modulatory capacity of aVNS to influence the parasympathetic activity seems to be the highest for the personalized systole-gated aVNS during inspiration. The personalised aVNS may favor consistency of therapeutic effects avoiding non-responders, may reduce side effects of aVNS, and minimize energetic footprints of stimulation patterns.

## Data Availability

The raw data supporting the conclusions of this article will be made available by the authors on justified request, without undue reservation.
